# Incidence and treatment of malignant tumors of the genitourinary tract in renal transplant recipients

**DOI:** 10.1590/S1677-5538.IBJU.2017.0471

**Published:** 2018

**Authors:** Juan Manuel Ochoa-López, Bernardo Gabilondo-Pliego, Sylvain Collura-Merlier, Jaime O. Herrera-Cáceres, Mariano Sotomayor de Zavaleta, Francisco Tomás Rodríguez-Covarrubias, Guillermo Feria-Bernal, Fernando Gabilondo-Navarro, Ricardo Alonso Castillejos-Molina

**Affiliations:** 1 Department of Urology, Instituto Nacional de Ciencias Médicas y Nutrición Salvador Zubirán, México

**Keywords:** Neoplasms, Urinary Tract, Kidney Transplantation

## Abstract

**Purpose:**

To provide data of the incidence and management of common urological malignancies in renal transplant recipients.

**Materials and Methods:**

We conducted a retrospective analysis of a prospective database from August 1967 to August 2015. A descriptive analysis of the sample was performed.

**Results:**

Among 1256 consecutive RTR a total of 88 patients developed malignancies (7%). There were 18 genitourinary tumors in the 16 patients (20.45 % of all malignant neoplasms), incidence of 1.27%. The most common neoplasm encounter was renal cancer (38.8%), followed by urothelial carcinoma (33.3%). Median follow-up of transplantation was 197 months (R, 36-336). Mean time from RT to cancer diagnosis 89±70 months (R, 12-276). CsA and AZA was the most common immunosuppression regimen in 68.75%. Mean follow-up after diagnosis was 103±72 months (R 10-215). Recurrence free survival rate of 100%. Overall survival of 89.5% of the sample; there were two non-related cancer deaths during follow-up.

**Conclusions:**

The incidence of neoplasms in RTR was lower than in other series, with favorable functional and oncologic results after treatment. This suggests that actions to reduce the risk of these malignancies as well as a strict follow-up are mandatory for an early detection and treatment.

## INTRODUCTION

Renal transplantation (RT) is a superior form of renal replacement therapy compared to dialysis, with significantly decreased mortality risk and improved quality of life in patients with end stage renal disease (ESRD) (1). In the current era of improved immunosuppression, patients have had significant improvement in graft and overall survival (2). Due to this rise in graft survival, a significant increase in the number of genitourinary post-transplant malignancies can be expected in the future. It is contemplated that within twenty years, death caused by tumor development will be the principal cause of mortality in this patient category (3).

Renal transplant recipients (RTR) have a risk three to five times higher than the general population of developing cancer, with lymphoma and skin cancer being the most predominant malignancies, followed by genitourinary tumors (4). This increment in incidence has been attributed to the decreased immune surveillance, activation of oncogenic viruses, chronic stimulation of immune system, immunosuppression, tobacco, and uremia (5).

In contrast to general population, cancer in RTR has a higher growth rate, is more aggressive, and tends to be multiple and disseminate early, both locally and systemically (6). Among urogenital tumors in RTR, a highly elevated incidence rate of RCC (15-fold) and bladder cancer (three-fold) has been estimated compared with non-transplanted population (7). Concerning testicular and prostate cancer, there is mix evidence, while some studies report an increased incidence for testicular (three-fold) and prostate cancer (PCa) (two-fold) (7), others show similar or lower incidences when compared to general population (7, 8).

The development of malignancies markedly decreases the survival rate of affected patients (4). Therefore, the aim of this study is to provide data of the incidence and management of common urological malignancies in RTR at our Institution.

## MATERIALS AND METHODS

We conducted a retrospective analysis of RTR at the Instituto Nacional de Ciencias Médicas y Nutrición “Salvador Zubirán from August 1967 to August 2015. A total of 1256 kidney transplants (717 male and 539 female) were analyzed. Of these, 939 were obtained from living donors and 317 from deceased donors. The median follow-up was 118±76 months.

Data collected included age, sex, time and type of dialysis, interval from renal transplant to cancer diagnosis, immunosuppression, smoking, location and type of tumor, treatment and outcomes.

A descriptive analysis of the sample was performed.

## RESULTS

Among 1256 consecutive RTR at our center, a total of 88 (7%) patients developed malignant neoplasms, of whom 16 (1.27%) had genitourinary tumors; 3 females and 13 males. There were 18 genitourinary tumors in the 16 patients (20.45% of all malignant neoplasm) (Table-1). Risk factors for the development of genitourinary tumors are shown in Table-2. The mean age at diagnosis was 35±15 (range, 17 to 70) years. The mean time from RT to cancer diagnosis was 89±70 (R, 12-276) months. For immunosuppression (Table-3), CsA (cyclosporine) was given in 11/16 (68.75%) patients, AZA (azathioprine) in 11/16 (68.75%), MMF (mycophenolate) 8/16 (50%), Tac (tacrolimus) 6/16 (37.57%), and PDN (prednisone) in 16/16 (100%) of the patients. Afterwards, immunosuppression doses were reduced in most cases. In 9 patients (56.25%), the Calcineurin inhibitor was interchanged for RAP (sirolimus) after cancer diagnosis.

**Table 1:** Distribution of genitourinary tumors.

Organ	Histopathology	n (%)	Treatment
	Renal cell carcinoma	7 (38.8)	Nephrectomy (2)
	- Clear cell in native kidney	5 (27.7)	Sunitinib (1)
	T1aN0MO, T4N1M1	2 (11.1)	Bilateral RN (1)
Renal	- Bilateral papillary type 1 in native kidney	2 (11.1)	
	T1aN0MO	3 (16.6)	PN (2)
	- Clear cell in kidney graft		RN (1)
	T1aN0M0 (3)		
Bladder	Urothelial carcinoma:	6 (33.3)	TURB (5)
	LG-NIMBC (5), Adenocarcinoma (1)	5 (27.7)	Nephrouretectomy (1)
	Upper tract urothelial carcinoma	1 (5.5)	Chemotherapy (1)
Prostate	Adenocarcinoma: 7(3+4), 7 (4+3)	2 (11.1)	RP (1) ADT (1)
Penis	Squamous cell carcinoma: In situ (2)	2 (11.1)	Circumcision (2)
GCT	Non-seminomatous germ cell tumor	1 (5.5)	Chemotherapy

**Table 2:** Risk factors for genitourinary tumors.

Risk Factors	Renal	Bladder	Penis	Prostate	Testes	Total
Age	36±17 (R 17-61)	29±8 (R 19-42)	38±10 (R 30-45)	64±8 (R 58-70)	25	**36±15 (R 17-70)**
Time of dialysis	31±33 (R 3-84)	26±15 (R 13-48)	19±12 (R 10-28)	38±19 (R 24-52)	35	**29±22 (R 3-84)**
Smoking	4/7 (57.14%)	2/6 (33.33%)	0/2 (0%)	1/2 (50%)	0	**8/16 (50%)**
Time of RT to caner	126±88 (R, 38-276)	37±23 (R,12-72)	156±68 (R 108-204)	41±2 (39-42)	48	**86±74 (R 12-276)**
Follow-up	42±37 (R 10-111)	167±72 (R 40-215)	121±43 (R 96-145)	76±6 (R 71-86)	176	**103±72 (R 10-215)**

**Table 3:** Immunosuppression.

	Renal	Bladder	Penis	Prostate	Testes	Total n (%)
CsA	3/6 (50%)	5/5 (100%)	2/2 (100%)	0/2 (0%)	1/1 (100%)	**11/16 (68.75%)**
AZA	3/6 (50%)	5/5 (100%)	2/2 (100%)	0/2 (0%)	1/1 (100%)	**11/16 (73.68%)**
MMF	3/6 (50%)	1/5 (20%)	1/2 (0%)	2/2 (100%)	0/1 (0%)	**7/16 (43.75%)**
Tac	3/6 (50%)	0/5 (0%)	0/2 (0%)	2/2 (100%)	0/1 (0%)	**5/16 (31.25%)**
RAP	4/6 (66.66%)	2/5 (40%)	2/2 (100%)	0/2 (0%)	1/1 (100%)	**9/16 (56.25%)**
PDN	6/6 (100%)	5/5 (100%)	2/2 (100%)	2/2 (100%)	1/1 (100%)	**16/16 (100%)**

In 42.10% of the cases, there was prior history of significant tobacco use. Mean follow-up after diagnosis was 103±72 (R 10-215) months. The recurrence free survival rate was 100%; none of the patients with localized tumors had recurrence during follow-up. There is one patient with metastatic RCC with no signs of clinical or radiological progression. The overall survival was 89.5%; there were two non-related cancer deaths during follow-up.

### Renal cancer

In 6 RTR, we found 7 kidney malignancies (38.8%) of all urological neoplasms detected). Of these, 5 were RCC (renal cell carcinoma) (27.7%); 2 patients developed clear cell carcinoma in native kidney (11.1%), one individual was found with synchronous bilateral papillary type 1 cancer in native kidney (11.1%), and 3 tumors were located in the renal graft (16.6%). The immunosuppression regimens of MMF/Tac/PDN with Daclizumab as induction were used in patients diagnosed with RCC of the renal graft. For individuals with RCC in native kidneys, the therapeutic agents used were CsA/AZA/PDN. Four patients were switched to RAP/PDN regimen. Radical nephrectomy was performed as definitive treatment in native kidney tumors, and in one non-functional renal graft with RCC.

Partial nephrectomy was conducted in two cases of kidney graft malignancies; histopathology revealed, clear-cell, stage pT1a, Fuhrman 2, RO-resection in both individuals. There were no significant changes in serum creatinine levels pre-op (0.8 mg/dL and 1.3 mg/dL) and post-op (1.0 mg/dL and 1.4 mg/dL), respectively.

All RCC were classified as low-grade T1a disease except for one case found at diagnosis as T4N1MI; a cytoreductive nephrectomy was performed followed by Sunitinib.

Clinically, 5 patients (83.3%) are in complete remission, and one (16.6%) in is partial response to Sunitinib after 10 months of follow-up, with no signs of clinical or radiological progression.

### Urothelial carcinoma

Urothelial carcinoma was the second most common urological malignancy. Six de novo neoplasms of the bladder occurred in 5 individuals (33.3% of the urological neoplasm detected). Five affected the bladder and one the graft's upper urinary tract.

Regarding bladder tumors, all were low-grade, non-muscle invasive, and treated with transurethral resection of bladder tumor (TURBT).

Concerning the patient with the graft upper tract urothelial carcinoma, an uncommon presentation of tumor in RTR, a nephroureterectomy of the renal allograft was performed. Histological analysis revealed a low grade urothelial carcinoma confined to the pelvis allograft, with lamina propria invasion and free surgical margins (T1G1).

No patient has had recurrence in a mean follow-up period of 167 months (R, 40-215). One patient died during TURBT due to bronchial aspiration; the histologic result in this case was a synchronous urothelial carcinoma and adenocarcinoma.

In all cases, the immunosuppressive treatment consisted of CsA/AZA/PDN; this combination was further changed after cancer diagnosis in two patients. Smoking was found in 2 of 5 patients (40%).

### Prostate cancer

Two patients received this diagnosis (12.5%), prostate specific antigen (PSA) screening was used as a screening tool. Histologically, both tumors were intermediate risk adenocarcinomas {Gleason 7 (4+3) and Gleason 7 (3+4)}, both clinical stages IIB (T2cN0M0, an T2bN0M0, respectively). The first patient underwent radical prostatectomy (RP). The second patient refused curative treatment (radical prostatectomy or radiotherapy). He decided to receive androgen deprivation therapy (ADT), despite informed metabolic and functional complications of this treatment. The mean follow-up was 76±6.36 months. The patient treated with ADT died 80 months after treatment of acute myocardial infarction owing to causes unrelated to cancer. The patient who underwent RP is disease free, with favorable biochemical control. The immunosuppression scheme was MMF/Tac/ PDN with Daclizumab as induction in both cases.

### Penis cancer

This malignancy was found in two patients (12.5%). Both were squamous cell carcinoma in situ of the prepuce skin treated with circumcision, with a mean follow-up period of 121±34.64 months and no recurrence during this period of time. The immunosuppression regimen was CsA/AZA/ PDN, both changed to Sirolimus after cancer diagnosis.

### Germ cell tumors (GCT)

One patient (6.25%) developed an extra-gonadal non-seminomatous GCT. The immunosuppression therapy was CsA/AZA/PDN, and shifted to Sirolimus after diagnosis. The patient was treated with four cycles of Bleomycin, Etoposide, Cisplatin, with a progression free survival of 176 months.

Regarding histocompatibility, we found that of those sharing 0-haplotypes, 47.3% developed malignancies, while of those with 1 and 2 haplotypes, the frequency of tumors was 36.8% and 15.7%, respectively.

Donor and recipient gender was analyzed, finding an overall donor-recipient gender mismatch of 68.4%. Interestingly, of those developing renal neoplasms, 100% had gender mismatch.

## DISCUSSION

This retrospective study comprises all de novo urological malignancies in RTR diagnosed over a period of 48 years. In our study, there were 16 patients with 18-de novo urological neoplasms (20.45% of all malignant neoplasms). We reported an incidence of urological malignancies after RT of 1.27%, which is lower than in other series. On average, it is estimated that the incidence of cancer in patients who have sustained kidney transplant is 3 to 5 times higher than that for the general population, and increasing with time. According to data published in other countries, the accumulated incidence of neoplasms can reach 7.5% at 3 years from RT (4), 20% at 10 years (3), and nearly 30% after 20 years (8). In our study, we observed a mean time from RT to cancer diagnosis of 89±70 months (R, 12-276), with shorter intervals for bladder, prostate and testis cancer (37±23, 41±2 and 48 months, respectively), and longer intervals for renal and penis cancer (118±79 and 156±68, respectively). This contrasts with published data that reported RCC is an early event occurring between 2 and 5 years after transplantation (9, 10), and others favoring a much later appearance (around 89 months) (11). This suggests that the risk begins from the early post-RT and remains present even after many years of RT.

In our study RCC was the most frequent tumor, representing 38.8% of all urological malignancies, similar to other series (11). The 3-year cumulative incidence in transplant recipients is reported by the USRDS to be 2.2%. More commonly, RCC arises from the native kidneys of transplant recipients, although conflicting data is found in literature (11, 12). These discrepancies are probably due to small sample sizes.

Since there is a clear growing incidence of neoplasms in RTR, the European Urological Association Guidelines on Renal Transplantation recommends that screening should be carried out annually for skin, lymphatic system and native kidneys, and for all other organs, the screening should follow the same rules as in the general population (13). In our Institution we performed a biannual screening for disease prevention, which has allowed us to detect 90% of renal tumors in early and localized stages.

If the RCC affects a non-functioning kidney (native or graft), treatment of choice should be radical nephrectomy; there is no clear consensus for treatment when RCC affects the graft. At our institution, RCC in the graft was detected in 3 patients; 2 of them with functional graft underwent partial nephrectomy, performed with good functional and oncological outcomes. No tumor recurrence or allograft failure was observed after a median follow-up of 32 months in these patients. Partial nephrectomy has been shown to be as effective as radical nephrectomy for cancer control and would be preferred in transplant recipients if technically feasible (14, 15), although other therapeutic techniques such as cryotherapy and radiofrequency ablation have been reported in T1a tumors with comorbidities with adequate short-term outcomes (16, 17).

Urothelial carcinoma is an uncommon tumor in RTR. USRDS reported a 3-year cumulative incidence of 0.66%, which accounts for 0.87% of all tumors in transplant recipients (4). However, the risk of developing urothelial carcinoma after RT is superior to in general population, and has been estimated at around 3 times higher. Furthermore, they tend to be more aggressive, poorly differentiated, rapidly progressive, and causing higher mortality in comparison to the general population (18). It is likely that the usual risk factors for urothelial carcinoma, e.g., smoking, adding to immunosuppression are the cause for this higher rate. We found 5 tumors in the bladder, and 1 in the pelvis of the graft (all low grade NMIBC). There are few reports in the literature about upper tract urothelial carcinoma affecting the renal allograft; conservative and radical treatments have been reported with good outcomes, arguing that proper treatments should be tailored according to each case in a multidisciplinary approach (19). Additionally, there was 1 case of adenocarcinoma of the bladder. The latter is an exceptional way of presentation of bladder carcinoma, with no cases reported in the literature in post RT patients. Also, contrary to the usual way of presentation of bladder tumors in RT recipients, in our series we observed all to be low grade, non-progressive or recurrent with an average follow-up of 167 months and with non-cancer associated mortality. All received immunosuppression regimen consisting of CsA and AZA; tobacco use was seen in 40%. The typical clinical presentation of bladder cancer in RTR has been previously reviewed, with painless hematuria being the most common trait, followed by dysuria, flank pain, and urinary obstruction (20). We performed in all RTR a strict biannual screening protocol including urinalysis, which helped us detect these malignancies in early stages.

The optimal treatment for patients with bladder carcinoma is not well defined. TURBT follows the same principles as in non-transplanted patients. Intravesical chemotherapy and immunotherapy has been used without added morbidity to RTR (21, 22); however, these need to be taken cautiously since the reported numbers are too small to draw solid conclusions. If technically feasible, surgical procedures, including cystectomy, are considered the treatment of choice.

We found 2 cases with intermediate risk prostate cancer (Gleason 7 (4+3) and Gleason 7 (3+4)). Some series of tumors in RTR reported higher incidences of prostate cancer than in general population (10, 23); in ours, there was a smaller incidence, perhaps owing to the younger age of our transplanted population (35±15 R, 17-70); this is similar to other series, that reported comparable or even smaller incidence, so there is no clear consensus on this issue (11, 24). Although screening for prostate cancer in RTR follows similar precepts than for the general population, we must consider prostate specific levels in patients with ESRD may be higher with an increased cancer detection rate than in non-transplanted individuals (25). Because transplant recipients have a reduced life expectancy, comorbid conditions should be assessed, including the current function of the transplanted organ, before considering screening. Surgical treatment for prostate cancer after kidney transplantation is technically challenging. However, there are series reporting open, laparoscopic, and robot assisted laparoscopic prostatectomy with adequate functional and oncological outcomes (26, 27). Literature regarding the use of radiation therapy is limited (26).

Penile cancer is an uncommon tumor in RTR. Nevertheless, this population has an excess risk of developing this type of neoplasms. There is a 2.41-fold increase in any human papillomavirus (HPV)-related cancer compared with population controls (25). We reported cases of in situ squamous cell carcinoma treated effectively with circumcision. There are no screening programs currently available for non-cervical HPV-related cancer. Therefore, early detection relies on clinicians and patient's awareness on early signs and symptoms of cancer.

In our series, a non-seminomatous GCT was reported. Standard chemotherapy was successfully applied without deleterious effects to the graft. Some studies show favorable outcomes with Cisplatin (28), but others have reported otherwise, mentioning nephrotoxicity if combined with CsA. This could be prevented by shifting the immunosuppressive regimen to RAP, or by replacing Cisplatin for Carboplatin (29).

The data in this study comprises a vast period of time in the era of renal transplantation; because of this, different immunosuppression schemes ranging from older to more novel, are included. Continuous improvements in the efficacy of anti-rejection drugs have greatly contributed toward prolonging the long-term survival of transplant recipients (30); however, life-long use of immunosuppressive drugs increases the risk of opportunistic diseases and malignancies. The frequency of cancer increases during immunosuppression. Around 10 years after continuous immunosuppressive therapy, approximately 20% of transplanted patients have been diagnosed with cancer, a risk 2- to 5-fold higher than in the general population (30). The duration of immunosuppressive therapy, the intensity of such, and the type of immunosuppressive agent, all have an impact on the development of post-transplant malignancy, rendering the immunosuppressive regimen an important risk factor requiring consideration. The role of particular immunosuppressive agents on the outcome of post-transplant neoplasms is controversial (31). The use of immunosuppressive schemes that combine a variety of agents makes it difficult to isolate the effects of individual agents on post-transplant malignancies, thereby limiting the data available. In our Institute, we found a more prevalent incidence of tumors with CsA/ AZA (68.75%) than with MMF or Tac (50% and 37.5%, respectively). In 9 patients (56.25%). the Calcineurin Inhibitor was exchanged for RAP after cancer diagnosis. Modification of the immunosuppressive regimen for RTR who developed tumors, is a matter of debate. Many studies have shown the beneficial immunosuppressive effect of RAP, as it has demonstrated an antitumor property with adequate outcomes (32-33). Thus, it should be considered for RTR presenting with malignancies.

Post-transplant mortality associated with de novo cancers is elevated-ten-year survival rate of 57%, as opposed to 93% in those without it. According to the USRDS, 7.5% of post-renal transplantation deaths are attributed to malignancy (4). Interestingly, in our study, we found an overall survival of 89.5%. There were two non-related cancer deaths documented during follow-up with a recurrence-free survival of 100% in a mean follow-up period of 103 months.

## CONCLUSIONS

The rising incidence of post-transplant malignancies poses a unique problem to this population. Because of the suitability for conventional and curative treatments, measures to reduce the risk of these malignancies and a strict follow-up are mandatory for an early detection and treatment of these. Management should be multidisciplinary in order to preserve graft function and obtain favorable oncological outcomes.
